# Emerging Roles of Adaptive Immune Response in Alzheimer's Disease

**DOI:** 10.14336/AD.2024.0564

**Published:** 2025-08-12

**Authors:** Cong Li, Qinghe Zhao, Lina Feng, Mingquan Li

**Affiliations:** ^1^College of Traditional Chinese Medicine, Changchun University of Chinese Medicine, Changchun, China.; ^2^Institute of Chinese Materia Medica, China Academy of Chinese Medical Sciences, Beijing, China.; ^3^Department of Neurology, the Second Affiliated Hospital of Shandong First Medical University, Shandong First Medical University & Shandong Academy of Medical Sciences, Taian, China.; ^4^Department of Neurology, the Third Affiliated Clinical Hospital of the Changchun University of Chinese Medicine, Changchun, China.

**Keywords:** Adaptive immune response, Alzheimer's disease, Neuroinflammation, T cells

## Abstract

Alzheimer's disease (AD) is the most common neurodegenerative disease and is characterized by progressive cognitive decline. Pathologically, this disease is associated with the accumulation of extracellular amyloid plaques, intracellular neurofibrillary tangles (NFTs), and neuroinflammation. Current drug treatments primarily focus on managing symptoms rather than stopping disease progression. Disease-modifying therapies target the clearance of amyloid plaques through active and passive immunity methods. Although successful in animal models, human trials have shown adverse effects, such as meningoencephalitis, in a small number of patients who received active immunity methods. The efficacy of active immunity methods in treating AD remains uncertain, but passive immunity methods amyloid-beta (Abeta)-specific monoclonal antibody therapies such as aducanumab and lecanemab have been approved by the FDA. Despite the limitations of immune-based therapies, T-cell, and chimeric antigen receptor-based treatments show promise, but new guidelines are necessary to address potential adverse events. Research into the relationship between adaptive immune responses and AD is expected to provide innovative treatment approaches.

## 1. Introduction

**Figure 1.**
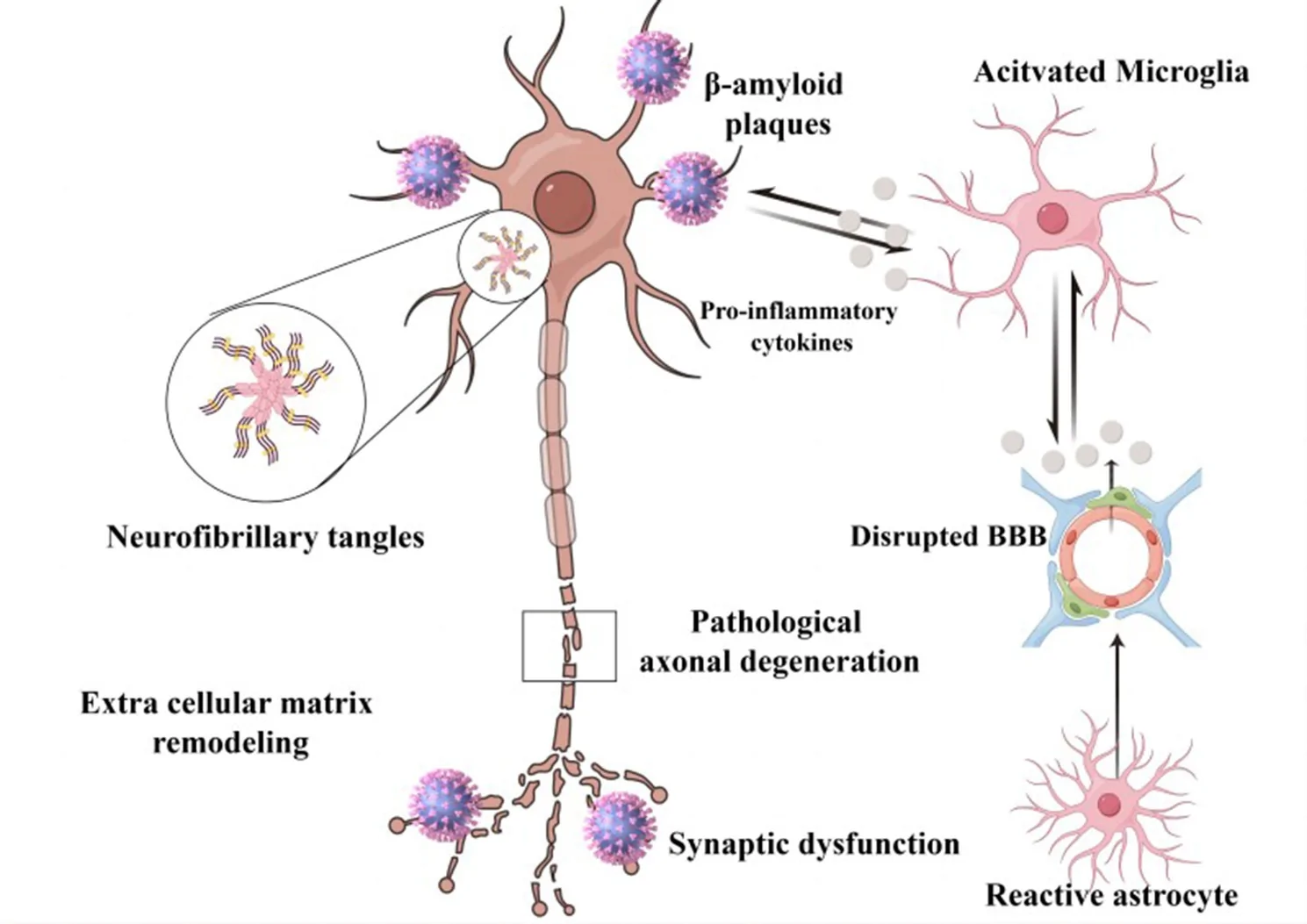
**Schematic diagram of pathogenesis and pathophysiological processes of AD**. The extracellular matrix (ECM) is a complex network dynamic structure made up of macromolecules secreted by cells into the extracellular interstitium. It consists of the interstitial matrix and basement membrane. ECM components not only contribute to maintaining tissue integrity, but also act as signaling molecules that play a role in various biological processes. Dysregulation of the ECM is a key factor in the development of many chronic diseases. From an immunological standpoint, the ECM also contains a variety of secreted proteins, such as cytokines, chemokines, and growth factors, which may be involved in regulating immune cell functions.

Alzheimer's disease (AD), a progressive neuro-degenerative condition, is characterized by a gradual decline in cognitive and behavioral functions. The pathogenesis of AD involves deposition of amyloid plaques, and formation of neurotoxic oligomers of the amyloid-beta (Abeta) peptide. This results in NFTs made up of the hyperphosphorylated microtubule-associated protein, Tau (p-Tau), neuroinflammation, neuronal and synaptic loss, and, ultimately onset of dementia [1-5]. The prevalence of AD has been on the rise, with its underlying causes and mechanisms still not fully elucidated. Neuroinflammation, an immune response triggered by glial cells in the central nervous system (CNS), is emerging as a significant pathological feature of AD, alongside Abeta deposition and NFTs (Fig. 1). This inflammatory process plays a crucial role in the onset and progression of AD and is closely linked to other neurodegenerative diseases, such as Parkinson's disease (PD), amyotrophic lateral sclerosis (ALS), and multiple sclerosis [6]. Immune activation in the CNS is a hallmark of neurodegenerative diseases, often resulting in neuronal damage. However, not all CNS immune responses are detrimental; some can actually aid in tissue and cell repair and regeneration in certain situations [7]. Microglia, for example, play dual roles in both causing damage and maintaining the dynamic balance of the brain in neurodegenerative diseases. Alongside microglia, T cells have the potential to contribute to recovery from neurodegenerative diseases due to intricate interactions within the neuroimmune system at the cellular and molecular levels. Immune cells not only release molecules that can harm nerves but also produce neuroprotective agents. However, the exact mechanism of action of these molecules remains unclear.

The immune response in brain tissue is tightly regulated by the CNS, which limits the entry of immune components and the activation of its own tissue. The CNS relies in part on the blood-brain barrier (BBB) for its immune properties. While the CNS is considered an immune-privileged environment, both innate and adaptive immune responses can be elicited. Innate immune responses are common in the CNS, but the presence of an active anti-inflammatory environment makes it challenging to initiate local adaptive immune responses. In certain neurodegenerative diseases such as AD, the exact impact of the adaptive immune response on neuronal damage and whether its activation is a secondary response to nerve damage remain unclear. This article reviews the mechanisms of the adaptive immune response in the CNS in AD, with the goal of suggesting new treatment strategies for this disease.

## 2. Current clinical research status of pharmaceutical treatments for AD

Current drug treatments for the AD dementia stage primarily consist of drugs aimed at improving cognitive symptoms, addressing behavioral and psychological symptoms of dementia (BPSD), and disease-modifying drugs. Approved medications to enhance cognitive symptoms include cholinesterase inhibitors (ChEIs) like donepezil, rivastigmine, and galantamine, as well as the N-methyl-D-aspartic acid (NMDA) receptor antagonist memantine. Additionally, mannate sodium capsule (GV-971) is a drug that targets the brain-gut axis for treatment [8].ChEIs have been shown to enhance cognitive function and daily living abilities in patients with AD, supported by level I evidence and a level A recommendation [9]. Additionally, memantine is recommended for the treatment of moderate to severe AD based on level I evidence and a level A recommendation [9]. GV-971 has demonstrated efficacy in improving cognitive function in patients with mild-to-moderate AD, supported by level II evidence and a level B recommendation [9]. It is important to individualize the treatment of BPSD, with non-drug interventions as the first-line approach. Drug treatment should only be considered when non-drug interventions are ineffective or when BPSD significantly impacts the patient's quality of life, or in cases of emergencies or safety concerns. When using antipsychotic drugs for symptomatic treatment, it is recommended to start at a low dose and gradually increase, based on level II evidence and a level B recommendation [9, 10]. Drugs utilized in the treatment of BPSD encompass CHEIs, NMDA receptor antagonists, atypical antipsychotics, typical antipsychotics, antidepressants, and mood stabilizers. Disease-modifying therapeutic drugs primarily function by enhancing the clearance of Abeta or tau proteins in the brain, which can potentially slow down the progression of the disease. These drugs are currently a key area of focus in AD drug research and development.

The availability of anti-Abeta monoclonal antibodies has sparked interest in anti-Abeta treatment within AD research. Therapies aim to reduce Abeta production (BACE-1 inhibitors and gamma-secretase inhibitors), prevent Abeta aggregation, and promote Abeta clearance [11] (through Abeta metabolism promotion or active/passive immunity methods against Abeta) [12]. Given that Abeta deposition causes irreversible nervous system damage by the time clinical symptoms appear, active Abeta immunotherapy is more suitable for early prevention than for treating high-risk AD patients [13]. Monoclonal antibodies against Abeta in passive immunity methods can interact with different forms of Abeta, including soluble, oligomeric, and fibrillar Abeta, leading to the activation of microglia and cytokines for Abeta clearance. Various monoclonal antibodies have been created to target different forms of Abeta, including Crenezumab, Donanemab, and Gantenerumab for oligomers, and bapineuzumab and solanezumab for monomers. Additionally, there are antibodies like eculizumab and lecanemab that focus on soluble fibrils, as well as aducanumab, which targets Abeta fibrils and soluble oligomers. In June 2021, aducanumab was approved for marketing by the US FDA, establishing a precedent for the approval of other disease-modifying therapies based on biomarker responses [14]. Subsequently, in January 2023, the phase III Clarity-AD study of lecanemab demonstrated a 27% reduction in cognitive decline and other functional impairments over 18 months, meeting the primary objective of the trial [15]. Furthermore, in May 2023, Lilly reported positive outcomes from the phase III trial of donanemab TRAILBLAZER-ALZ2, showing a significant deceleration in cognitive decline among patients with early symptomatic AD [9]. These drugs have demonstrated efficacy in experimental stages, highlighting the crucial role of the immune response in AD and its potential for future treatments.

Anti-Tau protein therapies focus on inhibiting Tau protein hyperphosphorylation and aggregation, enhancing the stability of the cellular microtubule system, and promoting Tau protein clearance [16]. Most of the therapies currently in clinical trials for promoting Tau protein clearance involve immunotherapy. These trials are still in the early stages of clinical research. Anti-Tau protein vaccines, which are based on active immunity methods, aim to stimulate the patient's immune system to produce antibodies against the pathogenic Tau protein. This is typically achieved by using synthetic peptides that mimic pathological Tau protein epitopes as antigens [17]. For instance, AADvac1 has demonstrated strong immunogenicity, with 98.2% of patients producing specific anti-Tau protein antibodies. It has been shown to significantly slow down brain atrophy in AD patients, improve cognitive impairment, and exhibit good safety and tolerability [18, 19]. Gosuranemab (BIIB092) displayed good safety and tolerability in phase I clinical trials and showed disease-modifying potential. However, the phase II clinical trial was terminated due to lack of efficacy (NCT03352557) [20]. Phase II clinical trials assessing the efficacy and safety of Tilavonemab (ABBV-8E12, NCT02880956) and Zagotenemab (LY3303560, NCT03518073) in patients with early AD, but the results are pending publication [21]. Semorinemab (RO7105705) exhibited good safety in phase II clinical trials, but did not show significant efficacy in patients with prodromal to mild AD [22]. Although these antibodies show promise, their effectiveness remains uncertain. Active immunity methods targeting the N-terminus of Abeta_42_, such as CAD106, ACC-001 with Abeta_1-7_, and ACI-24 with Abeta_1-15_ epitopes, can generate Abeta-specific antibodies without T-cell activation but may lead to aseptic meningitis [23, 24]. Both bapineuzumab and solanezumab faced setbacks in phase III clinical trials in 2012 and 2016, respectively [25]. Aducanumab stands out as the first monoclonal antibody endorsed by the FDA for AD treatment [26]. Demonstrating efficacy in reducing Abeta deposition in the cerebral cortex of AD patients, Aducanumab's impact is contingent on dosage and duration, showing potential in mitigating cognitive decline among those with mild AD or Mild cognitive impairment (MCI). Nonetheless, elevated concentrations may lead to amyloid-related imaging abnormalities (ARIA) with edema and effusion [26]. On January 6, 2023, lecanemab-irmb received accelerated marketing approval in the United States, becoming the second Abeta monoclonal antibody to receive global marketing approval following Aducanumab, while it carries risks of ARIA with edema and effusion and bleeding [27]. High-dose Crenezumab exhibits promising clinical therapeutic effects in mild AD patients [28, 29]. However, in phase III clinical trials, higher doses of Crenezumab have yet to demonstrate significant efficacy in individuals with prodromal AD [30]. In a phase II clinical trial, Donanemab notably reduced plasma pTau-217 and glial fibrillary acidic protein (GFAP) levels in early AD patients, leading to improvements in cognition and daily activity scores, albeit with ARIA with edema and effusion as the primary adverse event [31]. Therefore, further exploration is necessary to clarify the clinical benefit of the immune response in AD.

## 3. Interplay between innate and adaptive immunity response in the homoeostatic and AD brain

The term "immune privilege" has its roots in historical observations indicating that foreign tissues or tumors, when transplanted abnormally, could survive in the brain parenchyma without the need for immunosuppression, unlike in peripheral regions. Notably, tissues that were initially transplanted to peripheral sites and subsequently moved to the brain were rejected [32, 33]. While the brain parenchyma might not typically host many immune cells, it has been observed that following peripheral priming and changes to the CNS environment, non-resident immune cells can migrate into and persist in the brain parenchyma [34]. The entire CNS is structured into several compartments, including the parenchyma, the subarachnoid space which houses the choroid plexus and CSF, as well as the meninges and the skull bone marrow [35-38]. The immune-privileged status appears to be contingent and varies regionally.

It is currently acknowledged that both innate and adaptive immune system cells are found in the homeostatic CNS. Within a stable brain environment, microglia continuously monitor the brain's parenchyma. There exist minimal quantities of both adaptive and innate immune cells, including T cells, B cells, neutrophils, monocytes, and macrophages derived from them, which have the ability to enter the brain-especially the meninges and contribute to brain development and learning processes [39-44]. Recent research indicates that both adaptive and innate immune cells can access the dura mater through channels in the skull that link the skull's bone marrow to the dura [45, 46]. Consequently, in homeostatic states, immune cells can particularly reach the border zones of the brain and establish potential connections with cell populations within the parenchyma. In the context of various diseases, particularly neurodegenerative disorders, a range of intricate changes take place in both adaptive and innate immunity [47]. AD stands out as the most prevalent neurodegenerative condition. It has been over a hundred years since Alois Alzheimer documented a case of a 51 year old woman in 1907 who showed signs of confusion, memory impairment, and disorientation. Pathological features of AD include regional brain atrophy along with a loss of neurons and synapses, which likely contribute to various cognitive impairments [48]. Recent findings have indicated that AD is linked to protein aggregation involving two primary proteins [49]. Abeta builds up in extracellular Abeita plaques as well as in the cerebrovascular walls, resulting in cerebral amyloid angiopathy (CAA) [50]. On the other hand, the microtubule-associated protein tau predominantly aggregates in the cytoplasm of neurons and their processes in a post-translationally modified form, which includes hyperphosphorylation and other modifications. Tau aggregates are found in dystrophic neurites surrounding Abeta plaques, neuropil threads (NTs), and NFTs [51]. AD goes beyond just being a proteopathy; the accumulation of Abeta and tau leads to damage in synapses, neuronal processes, and the BBB. This sequence of events triggers the activation of reactive microglia and astrocytes, as well as the infiltration of specific peripheral immune cells into the CNS. As a result, even conditions that are not usually considered autoimmune diseases can alter the immune-privileged environment, since changes in the local environment and the arrival of immune cells from the bloodstream and surrounding areas can induce noteworthy modifications in both the innate and adaptive immune systems within the brain's parenchyma in AD.

## 4. Adaptive immune response in AD

Adaptive immune responses are characterized by a high degree of specificity. This specificity arises from the extensive repertoire of antigen receptors found on T and B cells (T-cell receptor (TCR) and B-cell receptor (BCR)), which are the cellular components of the adaptive immune system [52]. The diversity of TCR and BCR is generated somatically through site-specific DNA recombination, with each receptor of a particular specificity being expressed clonally. Collectively, these receptors form a recognition repertoire that enhances the likelihood that the adaptive immune response can detect a wide array of antigens encountered throughout an individual's life. Consistent with the dual cellular composition of the immune system, immune responses are categorized into afferent and efferent arms. The afferent arm of the adaptive immune response involves the presentation of antigens to naive T cells, leading to their priming and activation. Soluble antigens and immune cells carrying antigens from the brain are transported to the deep cervical lymph nodes, where they are presented to naive T cells and B cells by professional antigen-presenting cells (APCs), such as mature DCs [53, 54].

More recently, research has highlighted the role of T cells in neurodegenerative diseases such as AD, PD, and ALS [55, 56]. Interestingly, certain alleles and variants of human leukocyte antigen (HLA) genes have been linked to an increased risk of developing AD and PD [56, 57]. HLA genes encode components of major histocompatibility complex (MHC) class I or class II, which are expressed by microglia - the innate immune cells of the CNS [56, 57]. This suggests that both microglia and T cells may interact in various ways to influence the inflammatory processes seen in neurodegenerative diseases [56, 57]. Neuroinflammation is a key feature of AD, and many studies have focused on intrinsic immunity rather than adaptive immunity. Recent research from a large-scale genome-wide association study revealed significant heritability enrichment in cell types related to both the innate and adaptive immune systems, particularly in AD. The strongest signals were from both the adaptive (e.g., T cells) and innate (e.g., CD14, a marker for monocytes, and CD15, a marker for neutrophils) immune systems in relation to AD [58]. The adaptive immune response encompasses the activation, proliferation, and differentiation of antigen-specific T/B lymphocytes into effector cells following antigenic stimulation. This process aims to effectively eliminate foreign substances in the body by distinguishing between 'self' and 'nonself', thus maintaining the relative stability of the internal environment. However, immune responses can sometimes lead to damage, resulting in hypersensitivity reactions or other immune-related diseases characterized as pathological immune responses. The primary components of adaptive immunity are lymphocytes and their products. There are two main types of adaptive immune responses: cell-mediated immunity, which is represented by T cells, and humoral immunity, which is primarily represented by B cells (with assistance from T cells). Both T and B lymphocytes originate from a common lymphoid progenitor in the bone marrow [56]. Within the thymus, T cells differentiate into two distinct functional subsets known as CD4^+^ and CD8^+^ T cells, named after their specific surface markers, CD4 and CD8 [56]. While there are other less common subsets of T cells, they are not in-depth discussed in this review.

A decline in immune competence and an increase in the incidence of autoantibodies are known to occur with age. In humans, changes in T cell subsets and reduced proliferative responses to mitogens have been documented with age. Additionally, alterations in the ratio of helper to suppressor T cell subsets have been observed in autoimmune diseases. In 1982, Macdonald et al [59]. examined peripheral blood from patients with AD, elderly normal subjects, and young normal subjects to analyze leucocyte phenotypes and proliferative responses to lectins. The lymphocyte count was depressed in the AD group. The monocyte count was reduced in the healthy aged and further reduced in the AD group [59]. Analysis of peripheral blood mononuclear cells (PBMCs) showed the proportion of T cells bearing the antigen detected by UCHT3 monoclonal antibody was reduced in the healthy aged and further reduced in the AD group [59]. Proliferative responses to PHA, Con A, PA, and PWM were similarly depressed in both the aged and AD groups [59]. In 1988, a study discovered that cells expressing leucocyte common antigen (LCA), along with T-cytotoxic-suppressor (T8) and T-helper-inducer (T4) antigens, were found in substantial quantities in the hippocampus and temporal cortex of brains affected by AD, as opposed to normal brain tissue [60]. Leucocytes and reactive microglia strongly expressed LCA, as well as the class II major MHC glycoprotein HLA-DR [60]. Further research has shown that HLA-DR immunoreactivity is typically limited in gray matter, with the exception of AD where it is found in association with nearly all neuritic plaques [61]. The presence of HLA-DR positive T cells has been identified in brain tissue affected by AD. Furthermore, cells resembling astrocytes have been found to express the natural killer cell antigen (Leu-11), and lymphocytes displaying T helper and T cytotoxic/suppressor cell antigens have also been observed [61]. In 2002, Togo et al. [62] conducted a study to examine the presence of T cells in the brain parenchyma of individuals with AD, non-AD degenerative dementias, and controls. Through semi-quantitative analysis of immunohistochemically stained tissue sections, the researchers observed T cells in all cases, with a higher number of T cells detected in the majority of AD cases compared to other cases [62]. The phenotype of T cells in the AD brain suggested that they were activated but not fully differentiated [62]. Through integrated analyses of multiple cohorts, one study identified both peripheral and central adaptive immune changes in AD [63]. Mass cytometry of PBMCs revealed an immune signature of AD characterized by increased numbers of CD8^+^ T effector memory CD45RA^+^ T_EMRA_ cells [63]. Subsequent analysis in a separate cohort showed a negative association between CD8^+^ T_EMRA_ cells and cognition [63]. Single-cell RNA sequencing revealed enhanced TCR signaling in these cells. Moreover, utilizing various single-cell TCR sequencing techniques in a third cohort, clonally expanded CD8^+^ T_EMRA_ cells were discovered in the cerebrospinal fluid of AD patients [63]. Laurent et al. [64] utilizing the THY-Tau22 mouse model, they investigated the progression of hippocampal tau pathology alongside cognitive decline and examined the connection between tau pathology and brain immune responses. In addition to the typical astroglial and microglial reactions, they discovered an infiltration of CD8^+^ T cells in the hippocampus of tau transgenic mice, accompanied by an early chemokine response, particularly involving CCL3 [64]. Interestingly, CD8^+^ lymphocyte infiltration was also observed in the cortex with frontotemporal dementia carrying the P301L tau mutation [64]. Furthermore, they conducted chronic depletion of T cells using an anti-CD3 antibody. This treatment successfully prevented T cell infiltration in the hippocampus of tau transgenic animals and reversed spatial memory deficits [64]. These findings collectively shed light on the role of the adaptive immune response in AD.

### 4.1 T cells and AD

The brain is a vital organ with active immunological properties, protected by both resident immune cells and those that infiltrate from peripheral sources [65]. Recent findings have highlighted changes in cells that reside within the brain, such as microglia, alongside peripheral immune cells like neutrophils, monocytes, T cells, and B cells. Moreover, there is growing evidence for interactions between innate and adaptive immune cells that contribute to the neuropathological development of AD [66-72]. It is now clear that, under stable conditions, the adaptive immune system is present in the brain. Although these adaptive immune cells, such as T cells and B cells, are generally found at low quantities, they can invade the brain's meninges, penetrate the dura mater through the skull's openings, and are vital for maintaining various brain functions, including neuronal support, brain development, and spatial learning [73]. This process is largely driven by cytokines produced by T cells, including interleukin-4 (IL-4), IL-17, and IFN-gamma [74]. Studies indicate that a lack of IL-4-secreting T cells in the meninges or an overabundance of these cells in the choroid plexus with aging negatively impacts brain health, emphasizing the role of the adaptive immune system in preserving brain function during stable conditions [75, 76]. A recent investigation into the heightened clonal expansion and increased activation of cytotoxic CD8^+^ T cells in the brains of individuals with mild MCI and AD underscores the potential roles these T cells may play in modifying disease outcomes in AD. Nevertheless, it remains unclear if cytotoxic T cells contribute to the pathogenic processes in patients with AD. Another consideration is that under disease states like AD, subsequent injury to the BBB resulting from pathological alterations could facilitate the infiltration of CD8^+^ T cells from the bloodstream and surrounding areas into the brain tissue, leading to further clonal expansion of TCR. However, the extent to which peripheral CD8^+^ T cells are capable of crossing the BBB and infiltrating the brain parenchyma in AD remains largely unknown. In addition, a decrease in circulating IFN-gamma-secreting T cells has been correlated with cognitive decline associated with aging in mouse models [77]. Likewise, reduced plasma levels of IFN-gamma in AD patients have been linked to the advancement of cognitive impairment [78].

Genome-wide association studies (GWAS) have identified various genes that may play significant roles in both adaptive and innate immune responses. Among these, *CD33* was the first gene linked with AD through a family-based GWAS [79]. Subsequently, researchers have pinpointed additional crucial AD-related genes, such as *TREM2, INPP5D, CLU, CR1, SPI1, ABCA7, EPHA1*, and the *MS4A* gene cluster [5, 80-83], all of which are associated with immune system functions. Notably, *CLU* and *CR1* are recognized as key risk factors for AD and are integral components of the complement pathway. *CR1* is instrumental in activating the complement system, facilitating microglial activity, and enhancing the phagocytosis of immune complexes, cellular debris, and Abeta [84-86]. Yet, the precise role of *CR1* in the development of AD-related neuropathology remains unclear. GWAS findings suggest that variants in the *CR1* gene may result in loss of function (LOF), potentially leading to diminished Abeta clearance from the periphery by erythrocytes and an imbalance in the complement system, including its inflammatory effects [85, 86] (Table 1).

A recent investigation revealed P-tau levels in the inferior parietal lobe, middle gyrus, and medial frontal gyrus showed a positive correlation with T-cell infiltration in AD patients[87]. In a study on APPswe/PSEN1dE9 mice, the downregulation of genes involved in synaptic plasticity, postsynapses, and glutamatergic synapses was observed when T cells infiltrated brain tissue, indicating a potential impact on neuronal activity and cognitive function [102]. Carriers of the apolipoprotein E4 (APOE4) allele, a major genetic risk factor for late-onset AD [103], displayed the highest T-cell infiltration in the hippocampus among different genotypes [88]. APOE is expressed in various organs, with particularly high protein levels observed in the liver and brain. In the brain, APOE is predominantly synthesized by astrocytes and oligodendrocytes under homeostatic conditions; however, its expression is significantly elevated in reactive microglia during disease states. APOE plays a significant role in brain lipid metabolism, influencing the lipid metabolism of both astrocytes and microglia. This modulation may be pertinent to certain aspects of its effects on AD pathology [104, 105]. Telomeres are small segments of DNA-protein complexes located at the ends of linear chromosomes in eukaryotic cells. These short telomeric repeats, along with telomere-binding proteins, create a unique 'cap' structure that plays a crucial role in preserving chromosome integrity and regulating the cell division cycle. A notable association has been observed in AD patients, linking T-cell telomere length with MMSE scores [89]. Additionally, PBMCs of AD patients exhibit shorter telomeres, with T-cell telomere length inversely related to the levels of the proinflammatory factor TNF-alpha, CD8^+^ T cells lacking CD28 expression, and apoptosis [89]. Compared with those of controls, the peripheral blood of AD AD model mice has a greater percentage of CD4^+^/CD8^+^ T cells, accompanied by lower CD8^+^ T-cell counts and reduced total CD3 expression [106, 107]. Peripheral blood analyses in AD patients revealed increased numbers of IL-2R^+^, HLA-DR^+^, CD25^+^, and CD28^+^ T cells compared to those in controls, indicating an immune response in the peripheral nervous system [90]. Additionally, studies have consistently reported higher levels of CD4^+^ and CD8^+^ T cells in the brain parenchyma and CSF of AD patients, with CD8^+^ T cells surpassing CD4^+^ T cells in absolute numbers. Both T-cell subtypes displayed the CD45RA^+^ and CD45RO^+^ phenotype, suggesting the presence of activated and potentially cytotoxic infiltrating T cells [64, 88, 108]. These findings suggest a close association between T cells and the development of AD.

**Table 1:** Extract from different sites in AD patients, their pathogenesis, and potential immune interactions.

NO.	Extract from different sites in AD patients	Disease-modifying role or correlation	Refs.
**1**	Peripheral blood from patients with AD, elderly normal subjects, and young normal subjects	Analysis of peripheral blood mononuclear cells (PBMCs) showed the proportion of T cells bearing the antigen detected by UCHT3 monoclonal antibody was reduced in the healthy aged and further reduced in the AD group.	[59]
**2**	The hippocampus and temporal cortex in the brains of individuals with AD compared to those in normal brain tissue.	Expressing leucocyte common antigen (LCA), along with T-cytotoxic-suppressor (T8) and T-helper-inducer (T4) antigens in the hippocampus and temporal cortex of brains affected by AD.	[60]
**3**	The hippocampus and temporal cortex in the brains of individuals with AD compared to those in normal brain tissue.	HLA-DR immunoreactivity is found in association with nearly all neuritic plaques in AD patients. The presence of HLA-DR positive T cells has been identified in brain tissue affected by AD.	[61]
**4**	Brain parenchyma of individuals with AD, non-AD degenerative dementias, and controls.	T cells in all cases, with a higher number of T cells detected in the majority of AD cases compared to other cases.	[62]
**5**	Performed mass cytometry of PBMCs.	1.Immune signature of AD that consists of increased numbers of CD8^+^T effector memory CD45RA^+^(T_EMRA_) cells. 2.CD8^+^T_EMRA_ cells were negatively associated with cognition.	[63]
**6**	Using single-cell TCR sequencing in the CSF of patients with AD.	They discovered clonally expanded CD8^+^ T_EMRA_ cells in the CSF of patients with AD.	[63]
**7**	Investigated the brains of 28 non-immunized patients with AD and 11 patients with AD immunized against Abeta_42_ (AN1792).	A recent investigation revealed P-tau levels in the inferior parietal lobe, middle gyrus, and medial frontal gyrus showed a positive correlation with T-cell infiltration in AD patients	[87]
**8**	Postmortem hippocampi and mid frontal gyrus samples of AD patients (Braak stage V-VI) and nondemented control subjects.	1.AD hippocampi harbored significantly increased numbers of extravascular CD3^+^ T cells compared to nondemented controls. 2.CD3^+^ T cells significantly correlated with tau pathology but not with amyloid plaques in AD samples.	[88]
**9**	An ongoing, unrelated study with AD, consisted of 15 AD patients and 15 healthy controls (HCs).	Telomere length of T cells, but not of B cells or monocytes, correlated with AD disease status, measured by Mini Mental Status Exam (MMSE) scores.	[89]
**10**	Examined PBMCs from 51 AD patients (29 with mild and 22 with moderately severe dementia) and 51 age-matched HCs.	1. A significant decrease in circulating B and CD8^+^CD28^-^ cells. 2.An increase in CD8^+^ cells expressing CD71^+^ and CD28^+^ in AD patients. 3.A significant decrease in IL-10 production after stimulation of PMBC with Abeta.	[90]
**11**	The gene expression of approximately 10,000 full-length genes was compared in mild/moderate dementia cases to non-demented controls that exhibited high AD pathology.	1. Compared to non-demented high-pathology controls, the hippocampus of AD cases had increased gene expression of MHC II and inversely correlated with cognitive ability. 2.The mild/moderate AD dementia exhibited decreased number of T cells in the hippocampus and the cortex compared to controls.	[91]
**12**	Investigated the population structure of live microglia purified from human cerebral cortex samples obtained at autopsy and during neurosurgical procedures.	1. Microglial cluster 7, enriched genes depleted in the cortex of individuals with AD. 2.A decrease in the number of microglia expressing CD74 has been observed	[92]
**13**	Plasma samples from patients with AD (n=20),MCI with AD profile (n=20) and healthy control subjects (n=20).	1. Higher levels of IL-10, IL-1beta, IL-4 and IL-2 in both MCI groups, while there was no significant difference in inflammatory markers between dementia groups and controls. 2.for the first time that in AD, increased peripheral inflammation occurs early at the MCI disease stages.	[93]
**14**	Moderate-to-severe AD and in comparison, to healthy adults.	1. The Th1/Th2 ratios in AD patients were significantly higher than the HCs, 2.Significant relationships were noted between the changes in Th1/Th2 ratios with cognitive assessments.	[94]
**15**	38 patients had a diagnosis of AD, 34 patients had a diagnosis of MCI, 40 individuals were healthy.	1. A significant increase in IL-21 and IL-9 producing Abeta-stimulated CD4^+^ T cells, as well as in IL-23 and IL-6producing monocytes and CD4^+^ T cells that express the ROR-gamma and NFATc1 transcription factors. 2.The population of IL-10-producing monocytes was found to be decreased.	[95]
**16**	Analyzed neutrophil phenotypes and functions in 42 patients with AD (16 with MCI and 26 with dementia), and compared them to 22 age-matched healthy subjects	The homeostasis of circulating neutrophils in patients with AD and dementia has changed: the ratio of the harmful hyper reactive CXCR4^high^/CD62L^low^ senescent neutrophil subset to the CD16^brigh^t/CD62L^dim^ immuno-suppressive neutrophil subset increased in the later stages of the disease.	[96]
**17**	AD patients and healthy subjects	A significant decrease in the total (CD4^+^/CD25high/CD127low-neg) and resting (CD45RApos/CD25dim) Tregs in AD patients when compared to healthy subjects.	[97]
**18**	30 HC subjects, 26 patients with MCI, 30 patients with mild probable AD-related dementia, and 28 patients with moderate-to-severe probable AD-related dementia	1. The percentage of Tregs in the blood of patients with MCI was found to be the highest. 2.The level of TGF-beta in patients with MCI was significantly elevated. 3.Positive correlations were observed between the percentage of Tregs, IL-35, and MMSE in patients with MCI and probable AD-related dementia.	[98]
**19**	Immunization with Abeta peptide has been initiated in a randomised pilot study in AD.	Inflammatory infiltrates were composed of CD8^+^, CD4^+^, CD3^+^, CD5^+^ and, rarely, CD7^+^ lymphocytes, whereas B lymphocytes and T cytotoxic cells CD16, CD57, TIA and graenzyme were negative.	[99]
**20**	An AD tissue microarray with 2,325 tissue specimens from 3 defined CNS regions of 48 AD patients and 48 age-matched control patients.	The majority of neuritic plaques are characterized by the presence of IgG. In AD patients exhibiting prominently IgG-labeled neuritic plaques, there is a significant reduction in plaque burden accompanied by an increase in phagocytic microglia.	[100]
**21**	A case-control study among control individuals, late onset depression (LOD) and AD patients.	An increased CD4^+^ T cells expressing IL-10 were detected only in the AD group. There was no difference detected in allele or genotype analysis for IL-10 polymorphism among LOD, AD patients or controls.	[101]

### 4.2 CD4^+^ T cells in AD

T cells express molecules such as CD3, CD4, and CD8 on their surface, and CD3 is coexpressed by T cells. Mature T cells are categorized into naïve T cells, effector T cells, and memory T cells [109]. Effector T cells, also known as Teffs, are the final differentiated cells. These cells can be further classified into CD4^+^ T cells and CD8^+^ T cells based on the expression of CD molecules. CD4^+^ T cells participate in the immune response against foreign antigens, enhancing the binding of the TCR to MHC class II complexes. Current CD4^+^ T-cell subsets include Th1, Th2, Th9, Th17, Th22, Treg, and T follicular helper (Tfh) cells [110-112]. On the other hand, CD8^+^ T cells are involved in the immune response against self-antigens, enhancing the binding of the TCR to antigen-MHC class I complexes [113, 114]. MHC class I molecules are universally expressed on the surface of nucleated cells in organisms and play a crucial role in the presentation of self-antigens. In response to intracellular pathogens such as viruses, these proteins undergo changes, signaling to natural killer cells to eliminate the infected cell and initiate an immune response. On the other hand, MHC class II molecules are expressed only by specific immune cells, called APCs, such as macrophages, B cells, and dendritic cells. Recognition by MHC class II is vital for activating an adaptive immune response by interacting with lymphocytes such as CD4^+^ helper T cells. The increased infiltration of T cells into the brain fosters communication between T cells and microglia, leading to accelerated neuroinflammation, a process that relies on MHC class II [115].

In AD, the apoptosis of T cells has been linked to CD4^+^ T cells [116]. Multiple studies underscore the significance of effector and regulatory CD4^+^ T cells in the pathophysiology of AD [117]. Furthermore, they advocate for low-dose IL-2 treatment, which promotes the expansion and activation of regulatory CD4^+^ T (Treg) cells, as a promising therapeutic strategy for managing this condition [117]. Activated CD8^+^ T cells have neurotoxic effects and are known to induce significant neuronal death through mechanisms involving Fas ligand (FasL), lymphocyte function-associated antigen-1 (LFA-1), and cell-cell contact with CD40 [118]. A study revealed a positive correlation between the number of CD8^+^ T cells and MMSE score, as well as a negative correlation with CD4^+^ T cells in AD patients [119]. Subsequent research has shown that microglial activation and increased expression of MHC I and MHC II are observed in AD patients [120, 121], with a greater number of MHC II^+^ microglia found near AD-associated pathology [91]. Furthermore, T cells can trigger neuroinflammation in an MHC II-dependent manner [122]. An increased number of APCs expressing the MHC II-Abeta complex was also identified in a mouse model of AD [123].

CD74 is a crucial cellular component of dendritic cells that governs key immune responses in mammals. Dendritic cells possess distinct mechanisms for detecting and reacting to external dangers. Through experiments with CD74 knockout mice, Jefferies et al. [124] reported that CD74 plays a pivotal role in immune cells by linking the MHC I receptor to the site where the invading pathogen is isolated. A decrease in the number of microglia expressing CD74 has been observed in patients with clinically and pathologically confirmed AD [92]. Previous research has indicated a potential connection between key factors in AD and genes that encode HLA, a critical component of human adaptive immunity. HLA genes are involved in various immune response functions, including inflammation, T-cell transendothelial migration, infection, brain development, and plasticity, in AD pathogenesis [125]. Genes within the HLA-DR (MHC class II molecule) complex, specifically HLA-DRB1 and HLA-DRB5 [126], encode subunits of the MHC-II receptor [127]. Microglia selectively express Phospholipase C-gamma-2 (PLCG2). Genetic variants of PLCG2 have been shown to influence the phenotype and function of microglia, impacting the risk of developing AD. For instance, the loss-of-function variant PLCG2-M28L has been associated with increased AD risk, while PLCG2-P522R has been shown to mitigate disease progression in a mouse model of AD characterized by amyloid deposition. Additionally, SH2-containing inositol-5'-phosphatase 1 (SHIP-1) plays a role in hydrolyzing phosphatidylinositol 3-kinase (PI3K), a critical component of the phosphatidylinositol 3,4,5-triphosphate signaling pathway involved in T-cell immune responses. SHIP-1 is targeted by miR-155, a proinflammatory factor, in CD4^+^ T cells [128] and dendritic cells [129]. Studies have demonstrated that miR-155-deficient mice exhibit impaired dendritic cell antigen presentation and defective interactions between dendritic cells and T cells [130]. Patients with AD exhibit peripheral immune dysfunction, with CD4^+^ cells potentially impacting neuronal activity through infiltration of the BBB and interaction with glial cells in the brain [131]. Recent research by Machhi et al. [132] reported that CD4^+^ cells accelerate AD progression in mice, particularly through Teffs such as the Abeta-Th1 and Abeta-Th17 subtypes. These cells downregulate the function of regulatory T cells (Tregs) in both the peripheral nervous system and the CNS, leading to increased microglial activation and neuroinflammation.

#### 4.2.1 Th1 and Th2 in AD

The primary function of Th1 cells is to enhance cellular immunity and support the proliferation, differentiation, and maturation of Tc cells. Additionally, Th1 cells promote macrophage phagocytosis, enhance the cytotoxicity of natural killer (NK) cells, and inhibit the differentiation of Th2 cells. The secretion factors associated with Th1 cells include IFN-gamma, IL-2, and TNF-alpha. In a study focusing on Abeta-specific Th1, Th2, and Th17 cells and their impact on microglial activation in mixed glial culture, which mimics the various APC types found in the brain, researchers discovered that mixed glia effectively acted as APCs for Abeta-specific Th1 and Th17 cells [133]. In addition, the presence of Abeta-specific Th2 cells was found to inhibit the production of IFN-gamma by Th1 cells and of IL-17 by Th17 cells when glia serve as APCs [133]. Coculturing Abeta-specific Th1 or Th17 cells with glia resulted in a significant increase in the production of proinflammatory cytokines in response to Abeta, along with the upregulation of MHC II and costimulatory molecules on microglia [133]. Abeta-specific Th2 cells were found to suppress the production of IL-1beta and IL-6 by mixed glia stimulated by Th17 cells and decrease the expression of CD86 and CD40 on microglia induced by Th1 cells. Interestingly, the upregulation of MHC II and CD86 on astrocytes triggered by Abeta-specific Th1 and Th17 cells remained unaffected by Th2 cells [133]. These results indicate that Abeta-specific Th1 and Th17 cells play a role in promoting the inflammatory activation of glial cells, a process that is partially influenced by Th2 cells [133]. One study involved transferring T-cell-enriched populations of Abeta-specific immune cells from nontransgenic littermates into cognitively impaired APPswe/PSEN1dE9 [134]. The results indicated that limited reactivation of the Abeta-specific immune response could yield cognitive and pathological benefits for at least 2 months in these transgenic mice [134]. The recipients exhibited lower levels of soluble Abeta in the hippocampus, fewer plaque-associated microglia, and greater synaptophysin immunoreactivity than did the untreated controls. However, infusates enriched in Th1 cells or depleted of CD4^+^ T cells were found to be ineffective [134]. Self-antigens can trigger autoreactive effector T cells (Teffs) that promote proinflammatory and neurodestructive responses, ultimately leading to cognitive impairments [132]. In a study, cloned lines of Abeta-reactive Th1 and Th17 cells were developed and characterized to investigate their role in AD pathogenesis [132]. These Abeta-Th1 and Abeta-Th17 clones were transferred into APPswe/PSEN1dE9 mice, which exhibit characteristics of AD, resulting in accelerated memory impairment, systemic inflammation, increased amyloid burden, heightened microglial activation, and exacerbated neuroinflammation [132]. Both Th1 and Th17 Abeta-reactive Teffs were found to worsen AD pathology by suppressing anti-inflammatory and immunosuppressive Tregs in both the peripheral nervous system and CNS [132].

**Figure 2.**
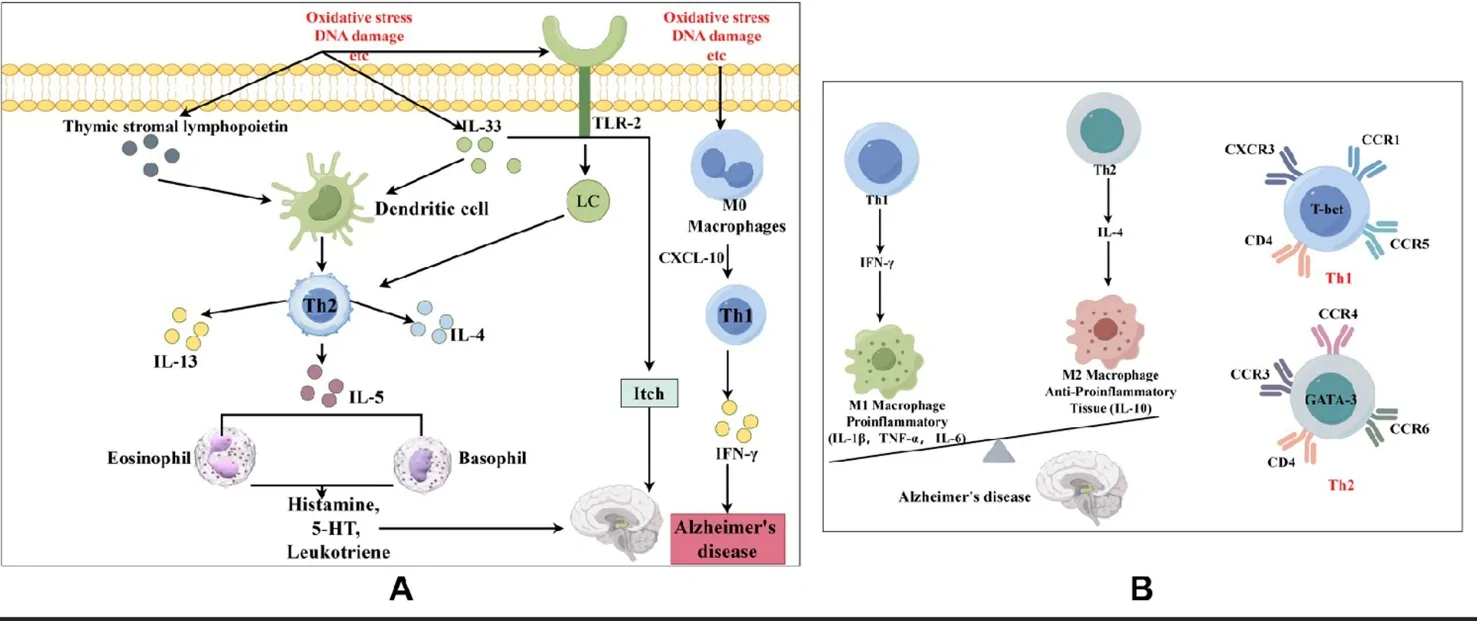
**The underlying mechanisms of action for Th1 and Th2 cells in AD, along with a schematic diagram illustrating the correlation between the imbalance of the Th1/Th2 ratio and the pathophysiology of AD**. (**A**) Various factors trigger the activation of Tfh, Th1, Th2, Th9, and Th17 cells, all of which are implicated in the pathogenesis of AD. (**B**) The balance of Th1-Th2 ratio plays a crucial role in maintaining the body's homeostasis. An imbalance in this ratio can result in the onset and progression of diseases like AD. It is commonly understood that Th1 and its secreted cytokines have pro-inflammatory effects, while Th2 and its cytokines have anti-inflammatory effects.)

IL-12 is a heterodimeric cytokine consisting of two subunits, p35 and p40. It has the ability to activate Natural killer cells (NK cells) and boost their cytotoxic function and the production of cytotoxic mediators. Additionally, IL-12 can stimulate the activation of naïve T cells, drive the differentiation of Th0 cells into Th1 cells, modulate the Th1/Th2 balance, and enhance the secretion of IFN-1, thereby enhancing macrophage activity [135]. Abeta-specific Th1 cells stimulate microglial activation and neuroinflammation through IFN-gamma production. Treatment with an IFN-gamma antibody can reverse the impact of Th1 cells on microglial activation and Abeta deposition [122]. AD is increasingly understood as a condition that also affects neurovascular function, manifesting as cerebral microbleeds and disturbances in autoregulation [136]. Autoregulation of cerebral blood flow, which is maintained at constant levels despite changes in perfusion pressure, is tightly regulated in the brain and leads to disturbances in AD [136]. The breakdown of autoregulation in AD may leave the brain vulnerable to cerebral microbleeds due to a diminished ability to control blood flow under increased pressure [136]. Studies have indicated that Abeta_1-42_ notably reduces contractility in human brain vascular smooth muscle cells (HBVSMCs), while Abeta_25-35_ enhances contractility. Notably, the inflammatory cytokine IFN-gamma also decreases the tonic contractility of HBVSM alone or in combination [136]. These findings suggest that the inflammatory environment in AD, along with the abundance of Abeta peptides, could contribute to autoregulatory dysfunction and increase the susceptibility of the brain to irregular blood circulation and microbleeds, which are key features of AD [136]. One study demonstrated that deletion of the IFN-gamma receptor specifically in Treg cells (encoded by Ifngr1) inhibits Th1-like polarization while promoting Th2-like polarization. TH1-like Treg cells were found to restrict CD8^+^ T-cell effector function, proliferation, and memory formation during both acute and chronic infections [110]. In the study by Schwartz et al. [137] utilizing mouse models of AD, immune checkpoint blockade directed at the programmed death-1 (PD-1) pathway induced a systemic immune response that relies on IFN-gamma. This response led to the influx of monocyte-derived macrophages into the brain. Activation of this immunological response in mice already exhibiting pathological results in the clearance of cerebral Abeta plaques and improvement in cognitive function [137]. Using AD mouse models, Geng et al. [8] reported that alterations in the gut microbiota composition during AD progression resulted in the peripheral accumulation of phenylalanine and isoleucine. These metabolites stimulate the differentiation and proliferation of proinflammatory Th1 cells. The presence of peripheral Th1 immune cells in the brain is linked to the activation of M1 microglia, which contributes to neuroinflammation in AD [8]. Monsonego et al. [138] reported that compared with MHCII- microglia, Abeta-Th1 cells polarized to secrete IFN-gamma and injected intracerebroventricularly (i.c.v.) into 5×FAD mice with AD induced the differentiation of MHCII^+^ microglia with distinct morphologies and improved plaque clearance capacity [138]. Interestingly, 5×FAD mice lacking MHCII exhibited increased amyloid pathology in the brain, heightened innate inflammation, and reduced phagocytic capacity [138].

Research on the mechanism of Th2 cells provides a promising avenue for developing treatments for AD. Currently, there are several preventive vaccines in development for AD. To delay the onset of the disease, the adjuvants used in these vaccines must trigger a Th2 immune response that is effective in reducing inflammation [139]. Achieving an effective immune response from a vaccine would require the coordinated delivery of immunogens to dendritic cells for priming, ultimately leading to polarized Th2 immunity [139]. This immune response should not only generate neutralizing antibodies against neurotoxic Abeta oligomers but also produce anti-inflammatory cytokines to combat the inflammation that worsens AD [139]. Th2 cells can secrete cytokines such as IL-4, IL-5, IL-6, IL-10, and IL-13. These cytokines support Th2 cell proliferation, aid in B-cell activation and promote B-cell proliferation, differentiation, and antibody production, contributing to humoral immunity. Additionally, these cytokines can suppress the proliferation of Th1 cells [140, 141]. In the context of neuroinflammation, astrocytes release TGF-beta, which serves to restrict microglial activation [142]. Astrocyte-derived TGF-beta and IL-33 can stimulate synaptic shear activity in microglia, potentially leading to aberrant elimination of neuronal synapses and alteration of brain circuits. Activated microglia may induce neurotoxic A1 state production in astrocytes by secreting IL-1-alpha and TNF-beta [143]. Th2 cells secrete the cytokines IL-4 and IL-10, which are neuroprotective against CNS nerve damage [144-146]. Alan et al. [93] demonstrated that higher concentrations of IL were found in the peripheral blood of patients with AD-type MCI, while IL-4 levels decreased with increasing disease severity. Another study revealed that patients with AD and MCI had significantly fewer IL-10-producing CD4^+^ T cells than HCs [93]. While John et al. [94] found that the Th1/Th2 ratios in patients with AD were significantly higher than those in HCs. Additionally, five of the six ratios (IL-2/IL-10, IFN-gamma/IL-10, IL-2/IL-4, IFN-gamma/IL-4, and IFN-gamma/TNF-alpha) decreased from baseline to the 12-month follow-up, with the exception of the IL-2/TNF-alpha ratio. Furthermore, several significant relationships were observed between changes in Th1/Th2 ratios and cognitive assessments [94]. The choroid plexus, an epithelial structure located in the brain's ventricles, plays a significant role in the formation of the blood-CSF barrier [147]. This structure is essential for maintaining the brain's homeostasis by releasing neurotrophic factors into the CSF, aiding in the clearance of Abeta, and facilitating the movement of leukocytes [147]. Research on naïve mice has revealed that over 50% of the T cells in the brain's choroid plexus stroma consist of CD4^+^ and CD8^+^ subsets [72]. Most of these T cells exhibit characteristics of effector memory phenotypes, including Th1, Th2, and Tregs, and possess the capability to detect antigens within the CNS [76]. However, in aged individuals, the choroid plexus shows an altered ratio of Th1 to Th2 cells, which results in heightened levels of the chemokine CCL11, diminished leukocyte permeability due to the differing effects of IL-4 and IFN-gamma on the choroid plexus epithelial cells, and impaired cognitive function [148].

Considering the unique and important functions of different T helper subsets in a range of diseases, it is crucial to comprehend how various Th cell populations specifically affect AD. In studies using APPswe/PSEN1dE9 models, the adoptive transfer of Abeta-specific Th1 cells, unlike Th2 or Th17 cell transfers, led to worsened AD pathology, heightened microglial activation, and compromised cognitive abilities [149]. The processes responsible for the plaque-clearing and pathogenic effects of Th1 cells were clarified when, after Abeta vaccination, Abeta-specific IFN-gamma-producing T cells penetrated the brains of J20 mice, resulting in plaque reduction while also producing meningoencephalitis, reminiscent of the pathogenicity connected with AN1792 [150]. Additionally, direct injections into the cerebrospinal fluid of Abeta-specific Th1 cells promoted neurogenesis and aided in the clearance of amyloid plaques without inducing autoimmunity, suggesting that peripheral Th1 cells have another function in APPswe/PSEN1dE9 mice models [151]. Conversely, the adoptive transfer of Abeta-specific Th2 cells, although showing no signs of brain infiltration, enhanced the working memory of APPswe/PSEN1dE9 mice and mitigated vascular amyloidosis and systemic inflammation [152]. Therefore, Abeta-specific Th1 and Th2 cells seem to affect the pathology of Abeta in various and distinct manners (Fig. 2).

#### 4.2.2 Th9 and AD

The cytokine IL-9, which is secreted by Th9 cells, is significantly elevated in patients with AD [95]. Th9 cells, a recently identified subset of CD4^+^ helper T cells, play diverse roles in various inflammatory immune disorders, oncologic diseases, and other conditions due to their production of IL-9 [153-156]. Under specific circumstances, various cell types can differentiate into Th9 cells, including Th2, Treg, and memory T cells. Conversely, Th9 cells can also transition into other helper cell types and secrete their respective factors, indicating a level of plasticity [157-159]. When TGF-beta was added to a Th2 cell line that initially did not produce IL-9, there was a notable increase in IL-9 expression, suggesting the induction of Th2 to Th9 cell transformation by TGF-beta, as proposed by Veldhoen et al. [156] and Tan et al. [160] observed that Th9 cells can exhibit Th1-, Th2-, and Th17-related characteristics and secrete significant amounts of IFN-gamma, IL-4, and IL-17 in a specific cytokine environment, although IL-9 was not detectable. Putheti et al. [161] demonstrated in human subjects that TGF-beta and IL-4 not only induced initial CD4^+^CD45RA^+^ T cells but also triggered the secretion of IL-9 from memory CD4^+^CD45RO^+^ cells. Additionally, Tregs can differentiate into Th9 cells. Liu et al. [162] reported that Th2 cells facilitate the conversion of Treg cells to Th9 cells, a process that can be suppressed by specific antibodies against IL-4 or TGF-beta. Kuchroo et al. [163] showed that IL-4 impeded the generation of TGF-beta-stimulated Foxp3^+^ Treg cells, leading to a shift toward the activation of T helper cells that produce IL-9 and IL-10. Subsequent research demonstrated that the combination of TGF-beta and IL-2 promoted the initial activation of CD4^+^ T cells, leading to the generation of Foxp3+ Treg cells with elevated expression of glucocorticoid-induced tumor necrosis factor receptor-related (GITR). Following the introduction of dopamine transporter-1 (DAT-1), a functionally related antibody, the Treg population decreased, with approximately 30% to 40% of Tregs transitioning into Th9 cells [164, 165]. This observation suggests a potential role for Th9 cells in modulating the development of AD through their plasticity, although further investigations are required to confirm this hypothesis.

#### 4.2.3 Th17 and AD

By utilizing a triple transgenic mouse model to simulate Abeta and tau neuropathologies, researchers observed heightened T and B lymphocyte activation, indicating the potential involvement of the adaptive immune system in AD pathology. Furthermore, analysis of cytokine levels revealed increased IL-2, TNF-alpha, IL-17, and GM-CSF concentrations, suggesting Th17 polarization [107]. In a separate study involving rats overexpressing APP, notable increases in IL-17, IL-22, and RORgammat were detected in the hippocampus, CSF, and serum [166]. Recent investigations also showed that treatment with anti-IL-17 antibodies and neutralization of the cytokine IL-17 led to enhanced cognitive function and reduced Abeta-induced neuroinflammation in adult mice. This was evidenced by decreased levels of Abeta_1-42,_ GFAP, S100 proteins, and myeloperoxidases (MPOs) [167], underscoring the potential collaborative role of IL-17 and its associated cytokines in exacerbating neuroinflammation and neurodegeneration in AD [168]. To further investigate inflammation in AD, Saresella et al. [95] conducted a comprehensive immunophenotypic and functional analysis of T lymphocytes stimulated by Abeta in AD patients, comparing the data to those of age-matched healthy individuals [95]. The results suggest that a significant increase in cytokines (IL-21, IL-6, and IL-23) and a transcription factor (RORgamma) is associated with the differentiation of Th-17 cells, as well as elevated levels of cytokines (IL-21 and IL-22) produced by these cells and increased levels of IL-9, which is generated by Th-9 cells, in AD [95]. Recent research in both human and animal models has additionally highlighted the significance of Th17 lymphocytes in the initial phases of AD [169].

Under the influence of TGF-beta alone, activated Th0 cells differentiate into Foxp3^+^ Treg cells. When both TGF-beta and IL-6 are present, Th0 cells differentiate into Th17 cells. Th17 cells primarily secrete IL-17A, IL-17F, and IL-22, with the main transcription factors being STAT3 and RORgammat, among others [169-172]. To explore the potential role of peripheral Th subsets in AD, Maler et al. [173] conducted a case-control study with 54 participants who were diagnosed with AD dementia, MCI due to AD, MCI unlikely due to AD, and HCs. A study revealed a notable increase in the number of CD3^+^CD8-IL-17A^+^-IFNgamma-Th17 cells in individuals with MCI-AD compared to those with MCI, which was not related to AD or HCs [173]. The increase in circulating CD3^+^CD8-IL-17A^+^-IFNgamma-Th17 cells in the initial phases of AD indicates a possible association between the adaptive immune system and neuropathological alterations in AD. This finding suggests a potential correlation between Th17 cells and the neuropathology and neurodegeneration observed in AD [173]. Elevated levels of IL-6 have been found in AD and MCI patients [174-176]. The cytokines IL-6 and IL-1beta play critical roles in the differentiation of Th17 cells [110]. Th17 cells, a subset of proinflammatory T helper cells, have been implicated in autoimmune and inflammatory conditions. Meta-analyses investigating cytokine alterations in AD patients have shown increased levels of IL-6 and IL-1beta in the bloodstream of AD patients and individuals with MCI [110]. Additionally, a meta-regression analysis in one of these studies revealed a relationship between lower MMSE scores and standardized mean differences in peripheral IL-6 levels between AD patients and individuals without cognitive impairment [110].

TGF-beta, a crucial regulator of the brain's response to injury and inflammation, has been associated with Abeta accumulation in living organisms. Research indicates that a modest increase in astroglial TGF-beta production in aged transgenic mice expressing the human beta-amyloid precursor protein results in a threefold reduction in the amount of parenchymal amyloid plaques, a 50% decrease in total Abeta burden in the hippocampus and neocortex, and a decrease in the number of dystrophic neurites [177]. Six weeks after receiving Th2-biased Abeta-specific T cells, mice with cognitive impairment demonstrated enhanced performance in working memory tasks [152]. This improvement was linked to elevated plasma levels of soluble Abeta [152]. Analysis of the hippocampus revealed a 30% decrease in plaque-associated microglia and reduced vascular amyloidosis in T-cell-treated mice[152]. Additionally, there was a decrease in the plasma levels of the inflammatory cytokines IFNgamma, TNF-beta, GM-CSF, IL-2, and IL-4, which are typically elevated in untreated APPswe/PSEN1dE9 mice[152]. Neutrophils extravasated and aggregated in areas with Abeta deposits in two transgenic models of AD (5×FAD and 3×Tg-AD mice)[96]. Within these regions, neutrophils release neutrophil extracellular traps (NETs) and IL-17[96]. Liu et al.[178] showed that IL-17 induces microglial activation, resulting in elevated expression of inducible nitric oxide synthase (iNOS). Activation of the STAT3-iNOS pathway, which relies on IL-17, was identified as essential for this mechanism[178]. Astrocytes play a critical role in maintaining CNS homeostasis and serve as a bridge between the CNS and the immune system. Qin et al.[179] examined the influence of IL-17 on the IL-6 signaling pathway in primary astrocytes. Their study revealed that IL-17 synergistically enhanced IL-6 expression in astrocytes, and this effect was mediated through various signaling pathways, such as the NF-кB, JNK, and p38 MAPK pathways[179]. However, several questions remain unresolved: Is the change in the ratio of Th17/Treg cells a cause or an effect of the alterations observed in AD? If the imbalance in the Th17/Treg ratio is solely a direct consequence of changes in the AD condition, it remains uncertain whether the increase in this ratio exacerbates AD, potentially leading to a vicious cycle, or whether it serves as a protective mechanism initiated by the inflammatory response that aids in the clearance of Abeta.

#### 4.2.4 Tregs and AD

Naïve CD4^+^ T helper (Th) cells and naïve Tregs (nTregs) undergo development in the thymus through positive and negative selection processes against self-antigens [180]. Naturally occurring nTreg cells exhibit heightened levels of Foxp3 and Helios transcription factors. Upon exiting the thymus, naïve CD4^+^ T helper cells differentiate into various subsets, such as Th1, Th2, Th17, or induced Treg (iTreg) subsets, based on polarizing cytokines and transcription factors [180]. Additionally, immunosuppressive Tregs can be induced in the periphery from Foxp3-Th cells in the presence of IL-2 and TGF-beta [180]. Over time, these cells acquire immunosuppressive markers similar to those of nTregs, along with sustained expression of the FoxP3 transcription factor[180]. Schwartz et al. [181] utilized the 5×FAD AD mouse model to show that temporary depletion of Foxp3^+^ Tregs or inhibition of their activity led to the clearance of Abeta plaques, a reduction in neuroinflammation, and improvements in cognitive function. They also found that this depletion affected the brain's choroid plexus, a key entry point for immune cells to the CNS, leading to the recruitment of immunoregulatory cells such as monocyte-derived macrophages and Tregs to areas of plaque buildup [181]. In a study by Marco et al. [97], an analysis was conducted to explore the differences and similarities in the Treg profile among individuals with AD. Tregs were identified as CD4^+^/CD25high/CD127low-neg cells using a novel flow cytometry technique and further categorized into resting (CD45RApos/CD25dim), activated (CD45RAneg/CD25bright), and secreting (CD45RAneg/CD25dim) cells [97]. The findings indicated a significant reduction in both total Tregs and resting Tregs in AD patients compared to healthy individuals [97].

Tregs are essential for maintaining immune tolerance and have known neuroprotective properties. However, their therapeutic potential is hindered by a lack of specific antigen recognition. In a study by Howard et al. [182], Tregs were engineered to express a TCR targeting the Abeta antigen derived from disease-specific T-cell effector clones. In an animal model of AD, these engineered Tregs showed promising results by reducing the Abeta burden, converting effector cells into regulatory cells, and reversing disease-related neurotoxicity [182]. Peripheral blood samples from patients with varying degrees of AD were analyzed to measure the percentage of CD4^+^CD25^+^CD127^low^/^-^ Tregs via flow cytometry, as well as the levels of IL-10, IL-35, and TGF-beta via ELISA [98]. The findings revealed positive correlations between Treg percentage, IL-35 levels, and MMSE scores in patients with MCI and probable AD-related dementia [98]. An Abeta vaccination trial (AN1792) in human AD patients halted prematurely due to the development of meningoencephalitides in 6% of patients, which is believed to be linked to the inappropriate activation of Abeta-specific T cells [99, 183]. On the other hand, while Abeta responses may help control Abeta plaque burden [100], evidence indicates that Abeta-specific CD4^+^ T-cell responses can actually reverse cognitive decline and synaptic loss in AD mice. Taken together, findings from both preclinical and clinical studies suggest that Abeta-specific T-cell responses have diverse functions with complex outcomes, which could be either harmful or beneficial, depending on how the reactivity and functionality of Abeta-specific Th cells are modulated [134, 152]. Guillaume et al. [184] investigated the impact of non-MHC genetic factors on CD4^+^ T-cell responses to Abeta. They found variations in the ability to generate Abeta-specific Treg responses among individuals, which significantly influences CD4^+^ T-cell reactions to Abeta in both normal and AD progression [184]. Lars et al. [185] This finding was further supported by linking Treg suppression to neurodegeneration in AD and PD patients. Disease severity was associated with the absence of Treg data, while a moderate correlation was noted between Foxp3 expression in AD patients and tau protein levels in the CSF [185].

The CD4 lymphocyte subpopulation and IL-2 cytokine level were significantly associated with the MMSE score in patients. CD4 lymphocytes showed a positive correlation, while IL-2 exhibited a negative correlation. Previous research by Nathalie et al. [186] demonstrated decreased IL-2 levels in the hippocampal biopsies of AD patients. In mouse models, IL-2 was shown to stimulate the expansion and activation of systemic and brain regulatory T cells [186]. Moreover, IL-2 in the hippocampus results in astrocytic activation, the recruitment of astrocytes around Abeta, a decreased Abeta_42/40_ ratio and Abeta plaque load, enhanced synaptic plasticity, and notably increased spine density [186]. A study by Alexander et al. [187] reported the significant role of IL-2R signaling-driven activation of STAT5 in improving the suppressive function of differentiated Treg cells [187]. Furthermore, IL-10 polymorphisms have been associated with AD. In a case-control study, Marco et al. [101] compared the associations between immune system phenotypes and genetic polymorphisms between control individuals and AD patients. Researchers have conducted real-time PCR for the IL-10 SNP (rs1800896) through genotyping analysis and assessed CD4^+^ T cells expressing IL-10 using flow cytometry [101]. These findings indicated an increase in CD4^+^ T cells expressing IL-10 specifically in the AD group, suggesting a potential role for IL-10 in modulating the immune response in AD patients [101]. In a recent study, researchers discovered that inhibiting GSK3 activity in human iTreg cells significantly improved their immunosuppressive function and prevented the formation of proinflammatory Treg cells [188]. Human iTreg cells typically produce low levels of IL-10, but inhibiting GSK3 led to a notable increase in IL-10 expression, promoting the generation of IL-10^+^ FOXP3^+^ iTreg cells [188]. Utilizing advanced cell sorting techniques, the team isolated IL-10-producing Treg cells, analyzed their transcriptomics and functional markers, and confirmed that IL-10^+^ FOXP3^+^ Treg cells exhibit enhanced immunosuppressive capabilities both *in vitro* and *in vivo* [188].

Anna et al. [189] reported that co-ligation of CD46 (membrane cofactor protein, MCP,CD46) and CD35 (complement receptor type 1, CR1) on activated CD4^+^ human T cells enhances CD25 expression, increases granzyme B production, and synergistically promotes cell proliferation. The study also confirmed the role of CR1 in Treg phenotype development by showing that its engagement enhances IL-10 production and reduces IFN-gamma release by activated CD4^+^ T cells in the presence of excess IL-2 [189]. Stanley et al. [190] conducted a study in which human Tregs were expanded *in vitro* for 24 days and administered to 5×FAD-Rag2^KO^ mice to establish an immunodeficient mouse model of AD. Changes in amyloid burden, microglial characteristics, and reactive astrocytes were assessed using ELISA and confocal microscopy [190]. NanoString Mouse AD multiplex gene expression analysis was used to investigate the impact of *in vitro*-expanded Tregs on the neuroinflammation transcriptome [190]. The results indicated that the administration of *in vitro*-expanded Tregs led to a reduction in the amyloid burden and reactive glial cells in the dentate gyrus and frontal cortex of 5×FAD-Rag2^KO^ mice. Analysis of inflammatory gene expression revealed the downregulation of proinflammatory cytokines, complement cascade components (C1qa, C1qb, C1qc, and C4a/b), Toll-like receptors (Tlr3, Tlr4, and Tlr7), and microglial activation markers (CD14, Tyrobp, and Trem2) following Treg administration [190]. Buyang-Soo et al. [191] developed a nanovaccine consisting of lipid nanoparticles loaded with Abeta peptides and rapamycin. This nanovaccine highlighted the combined effects of anti-Abeta antibody therapy and adoptive Abeta-specific Treg cell transfer. It effectively delivers rapamycin and Abeta peptides to dendritic cells, leading to the generation of both anti-Abeta antibodies and Abeta-specific Treg cells. Additionally, it cleared Abeta plaques in the brain, reduced neuroinflammation, prevented Th1 cell-mediated excessive immune responses, and inhibited cognitive impairment in mice [191]. These results underscore the potential of targeting Treg-mediated systemic immunosuppression as a therapeutic strategy for AD [181] (Table 2).

**Table 2:** Various AD models, their pathogenesis, and potential immune interactions.

NO	Various approaches for modeling AD	Disease-modifying role or correlation	Refs.
**1**	THY-Tau22 mouse model	1.An infiltration of CD8^+^ T cells in the hippocampus of tau transgenic mice, accompanied by an early chemokine response, particularly involving Chemokine C-C motif chemokine ligand 3 (CCL3). 2.CD8^+^ lymphocyte infiltration was also observed in the cortex.	[64]
**2**	Ablated the pool of CD8^+^ T cells in the blood, spleen and brain from 12 months-old APPswe/PSEN1dE9 and Wild Type mice for a total of 4 weeks using an anti CD8 antibody treatment.	CD8^+^ T cells infiltrate the aged and AD brain and that brain CD8^+^ T cells might directly contribute to neuronal dysfunction in modulating synaptic plasticity.	[102]
**3**	3×Tg-AD mouse model, which reproduces both Abeta and tau pathologies	Immunologic defects present in 3×Tg-AD mice (CD4/CD8 blood ratio; IL-5/IL-10 ratio in the cortex) and a modulation of CX3CR1^+^ cell population (in the bone marrow).	[106]
**4**	The triple transgenic mouse model (3×Tg-AD) to reproduce Abeta and tau neuropathologies.	1.Increased activation of both B and T lymphocytes. 2. A rise in serum IgG concentration, higher levels of IL-2, IL-17 and granulocyte-macrophage colony-stimulating factor.	[107]
**5**	Transgenic (tg) mice models of AD-like cerebral amyloidosis.	Increased numbers of infiltrating T-cells were found in amyloid-burdened brain regions of tg mice, with concomitant up-regulation of endothelial adhesion molecules Intercellular adhesion molecule 1 (ICAM-1) and Vascular cell adhesion molecule 1 (VCAM-1), compared to non-tg littermates.	[108]
**6**	Cultured microglia were pulsed with Abeta_1-42_ in the presence of CD40L and co-cultured with CD4^+^ T cells.	Microglia stimulate T cell-derived IFN-gamma and IL-2 production	[123]
**7**	Developed and characterized cloned lines of Abeta reactive type 1 T helper (Th1) and type 17 Th (Th17) cells, then adoptively transferred into APPswe/PSEN1dE9 mice.	An acceleration of memory impairment and systemic inflammation, an increase in Abeta burden, heightened microglial activation, and exacerbated neuroinflammation.	[132]
**8**	Generated Abeta-specific Th1, Th2, and Th17 cells and examined their role in modulating Abeta-induced activation of microglia in a mixed glial culture, a preparation which mimics the complex APC types in the brain.	1.Presence of Abeta-specific Th2 cells was found to inhibit the production of IFN-gamma by Th1 cells and of IL-17 by Th17 cells when glia serve as APCs. 2.Abeta-specific Th2 cells were found to suppress the production of IL-1beta and IL-6 by mixed glia stimulated by Th17 cells and decrease the expression of CD86 and CD40 on microglia induced by Th1 cells.	[133]
**9**	APPswe/PSEN1dE9 mice.	A single infusion of Abeta-specific immune cells derived from Abeta-vaccinated littermates significantly enhanced performance in cognitively impaired APPswe/PSEN1dE9 mice. Recipients exhibited reduced levels of soluble Abeta in the hippocampus, a decrease in plaque-associated microglia, and increased synaptophysin immunoreactivity compared to untreated controls.	[134]
**10**	5×FAD and APPswe/PSEN1dE9 mice.	Immune checkpoint blockade targeting the programmed death-1 (PD-1) pathway elicits an IFN-gamma-dependent systemic immune response, subsequently resulting in the recruitment of monocyte-derived macrophages to the brain.	[137]
**11**	Abeta-specific T helper 1 (Abeta-Th1 cells) T cells polarized to secrete IFN-gamma and intracerebroventricularly (i.c.v.) injected to the 5×FAD mouse model of AD	CD4 T cells induce a P2ry12^+^ MHCII^+^ subset of microglia, which play a key role in T cell-mediated effector functions that abrogate AD-like pathology.	[192]
**12**	APPswe/PSEN1dE9 mice	Significant infiltration of T cells in the brains of APPswe/PSEN1dE9 mice, and a proportion of these cells secreted IFN-gamma or IL-17.	[149]
**13**	Abeta-specific CD4 T cells generated by immunization with Abeta and a TLR agonist and polarized *in vitro* to Th1-, Th2-, or IL-17-producing CD4^+^ T cells, were adoptively transferred to APPswe/PSEN1dE9 mice.	1.Th1 cells, but not Th2 or IL-17-producing CD4^+^ T cells, increased microglial activation and Abeta deposition. 2.The effects of Th1 cells were attenuated by treatment of the APPswe/PSEN1dE9 mice with an anti-IFN-gamma Ab.	[149]
**14**	Amyloid precursor protein (APP)-transgenic (Tg) mice	1.A single immunization with the dominant T cell epitope Abeta_10-24_ induced transient meningoencephalitis. 2.Immune infiltrates were targeted primarily to sites of Abeta plaques in the brain and were associated with clearance of Abeta.	[150]
**15**	Wild-type C57BL/6 mice; Transgenic (Tg) APP/PS1 (APP-KM670/671NL, PS1-L166P) mice; APPswe/PSEN1dE9(APP-K594N/M595L, PS1-exon-9 deleted variant of presenilin 1) mice; IFN-gamma^KO^ mice; OT-II TCR Tg mice, and Tg mice expressing GFP under the direction of the human ubiqutin C promoter.	1.Th1 cells cross the ependymal layer of the ventricle and migrate within the brain parenchyma by stimulating an IFN-gamma-dependent dialogue with neural cells. 2.Abeta-specific Th1 cells target Abeta plaques, increase Abeta uptake, and promote neurogenesis	[151]
**16**	APPswe/PSEN1dE9 mice.	1.Pathological analysis of the hippocampus revealed a decrease in plaque-associated microglia and reduced vascular amyloidosis in T cell-treated mice. 2.The infusion of Abeta-specific Th2 cells led to a reduction in plasma levels of IFN-gamma, TNF-alpha, GM-CSF, IL-2, and IL-4, which are elevated in untreated APPswe/PSEN1dE9 mice.	[152]
**17**	Abeta_1-42_was bilaterally injected into hippocampus of rats to induce AD.	Th17 cells infiltrating the brain parenchyma in AD contribute to neuroinflammation and neurodegeneration through the release of pro-inflammatory cytokines and direct interactions with neurons via the Fas/FasL apoptotic pathway.	[166]
**18**	Abeta_1-42_was injected into cerebral ventricles of adult CD1 mice. Mice received IL-17Ab via i.c.v. either at 1 h prior to Abeta_1-42_injection or intranasal 5 and 12 days after Abeta_1-42_ injection	Pretreatment with IL-17Ab, administered i.c.v. or double intranasal, significantly reduced Abeta_1-42_ induced neurodegeneration, improved memory function, and prevented the increase of pro-inflammatory mediators in a dose-dependent manner.	[167]
**19**	hAPP/TGF-beta1 mice (Aged transgenic mice expressing the human beta-amyloid precursor protein (hAPP) ).	A modest increase in hAPP/TGF-beta1 mice results in a three-fold reduction in the number of parenchymal amyloid plaques, a 50% reduction in the overall Abeta load in the hippocampus and neocortex, and a decrease in the number of dystrophic neurites.	[177]
**20**	5×FAD and 3×Tg-AD mice	1.Neutrophils extravasated and were present in areas with Abeta deposits, where they released NETs and IL-17. 2.Neutrophil depletion or inhibition of neutrophil trafficking via LFA-1 blockade reduced AD-like neuropathology and improved memory in mice already showing cognitive dysfunction.	[193]
**21**	5×FAD mouse model	The transient depletion of Foxp3^+^ Tregs, or the pharmacological inhibition of their activity, leads to Abeta plaque clearance, a reduction in the neuroinflammatory response, and a reversal of cognitive decline.	[181]
**22**	TCR Abeta-Tregs were generated by CRISPR-Cas9 Knock Out of endogenous TCR and consequent incorporation of the transgenic TCR Ab identified from Abeta reactive Teff monoclones, then Adoptive transfer of TCR Abeta-Tregs to mice expressing a chimeric mouse-human amyloid precursor protein and a mutant human presenilin-1	Adoptive transfer of TCR Abeta-Tregs led to sustained immune suppression, reduced microglial reaction, and amyloid loads.	[182]
**23**	C57BL/6 (H-2^b^ haplotype), BALB/c (H-2^d^), SJL/J (H-2^s^), DBA/1 (H-2^q^), CBA/J mice (H-2^k^), C57BL/6 mice congenic for the H-2^s^ haplotype and APPswe/PSEN1dE9 mice (Thy1-APP^KM670/671NL^; Thy1-PS1^L166P^) on the C57BL/6 background.	The magnitude of Abeta-specific CD4^+^ T cell responses is critically controlled in both physiological and pathological settings by MHC-independent genetic factors that determine the overall potency of Abeta-specific Treg responses.	[184]
**24**	APPswe/PSEN1dE9 mice.	Within the hippocampus, IL-2 prompted astrocytic activation and the recruitment of astrocytes to areas surrounding amyloid plaques. This response was associated with a decreased Abeta_42/40_ ratio and a reduction in amyloid plaque load, as well as improvements in synaptic plasticity and a significant increase in spine density.	[186]
**25**	An immunodeficient mouse model of AD was generated by backcrossing the 5×FAD onto Rag2^KO^ mice (5×FAD-Rag2^KO^). Human Tregs were expanded *ex vivo* for 24 days and administered to 5×FAD-Rag2^KO^.	1.The elimination of mature B and T lymphocytes, as well as natural killer cells, in 5×FAD-Rag2^KO^ mice was associated with the upregulation of 95 inflammation-related genes. 2.Furthermore, the administration of *ex vivo* expanded Tregs resulted in a reduction of amyloid burden and reactive glial cells in both the dentate gyrus and frontal cortex of 5×FAD-Rag2^KO^ mice.	[190]

### 4.3 CD8^+^ T cells and AD

The TCR is a protein complex found on the surface of T cells that is responsible for recognizing specific antigens and molecules involved in immune responses. The TCR gene sequence is highly diverse due to intracellular rearrangement mechanisms, making it one of the most variable regions in the genome. This diversity plays a crucial role in how the human immune system adapts to different environmental challenges. When the TCR binds to MHC class I proteins on thymocytes, it initiates the positive selection of CD8^+^ T cells and leads to their differentiation into mature cytotoxic T lymphocytes[56]. Changes in peripheral blood cell profiles are commonly observed in individuals with inflammation and immune dysfunction, such as those with AD or MCI. Wang et al. [194] conducted a thorough meta-analysis to explore the relationship between peripheral blood cell counts and indices in patients with AD or MCI. The results suggest that variations in peripheral blood cell profiles, particularly in leukocyte, lymphocyte, neutrophil, and CD8^+^ T-cell counts, as well as the neutrophil-to-lymphocyte ratio (NLR) and the CD4/CD8 ratio, are closely associated with AD [194]. A growing number of immune-mediated CNS disorders with autoimmune origins have been identified, with neurons being the primary target of the immune response. Research indicates that cytotoxic CD8^+^ T cells play a key role in mediating neuronal damage in neurodegenerative diseases [195]. Doublecortin (DCX)^+^ cells located near plaques are not neurogenic, as they predominantly express markers for microglia (ionized calcium-binding adapter molecule 1) and phagocytosis (CD68 and TREM2) [196]. DCX is commonly utilized as a marker for immature neurons, and these cells were observed in proximity to Abeta plaques in various transgenic amyloid mouse models and human brains with plaque pathology [196]. Furthermore, a subset of DCX^+^ cells associated with plaques did not express ionized calcium-binding adapter molecule 1 but exhibited high levels of the pan-leukocyte marker CD45. These cells were identified as CD3 and CD8 positive T cells but lacked CD4 expression [196].

Inflammatory disorders affecting the CNS often result in synaptic loss, a process thought to involve phagocytic microglia and complement components. However, the specific mechanisms underlying abnormal synaptic connectivity in cases of neuronal damage caused by CD8^+^ T cells are not well understood. In a study using a murine model of encephalitis induced by CD8^+^ T cells targeting antigenic neurons, researchers analyzed the neuronal translatome [63]. They discovered that in this severe autoimmune disease driven by CD8^+^ T cells, neuronal STAT1 phosphorylation and CCL2 expression were closely linked to infiltrating CD8^+^ T cells and phagocytes [63]. In recent years, the importance of changes in peripheral lymphocytes in age-associated neurologic diseases has become increasingly apparent. Anne et al. [197] investigated whether changes occur in subsets (CD4^+^ and CD8^+^ cells) with aging. The phosphorylation of tyrosine residues decreases with age in CD4^+^ cells [197]. Decreased numbers of CD69 positive cells after 4 hours of mitogenic activation, altered expression of cytokines (IL2, IFN-gamma, and TNF-alpha), and reduced proliferation were observed during aging [197]. Furthermore, it was demonstrated that CD8^+^ lymphocytes respond more effectively to mitogenic stimulation in terms of CD69 expression and proliferation in both age groups (60 years old) [197]. These findings suggest that T-cell activation, influenced by TCR engagement, is significantly impaired in aging and affects both subsets [197]. The accumulation of senescent cells in the brain has been demonstrated to worsen pathological impairments in AD mouse models [198, 199]. The aging immune system is believed to exacerbate the pathological progression of AD. A recent study revealed that Tfam deficiency in T cells led to inflammation, causing multiple health issues and premature senescence, which included neurological impairments [200]. Although the authors did not directly investigate AD-related changes, they did observe neurodegenerative alterations [200].

**Figure 3.**
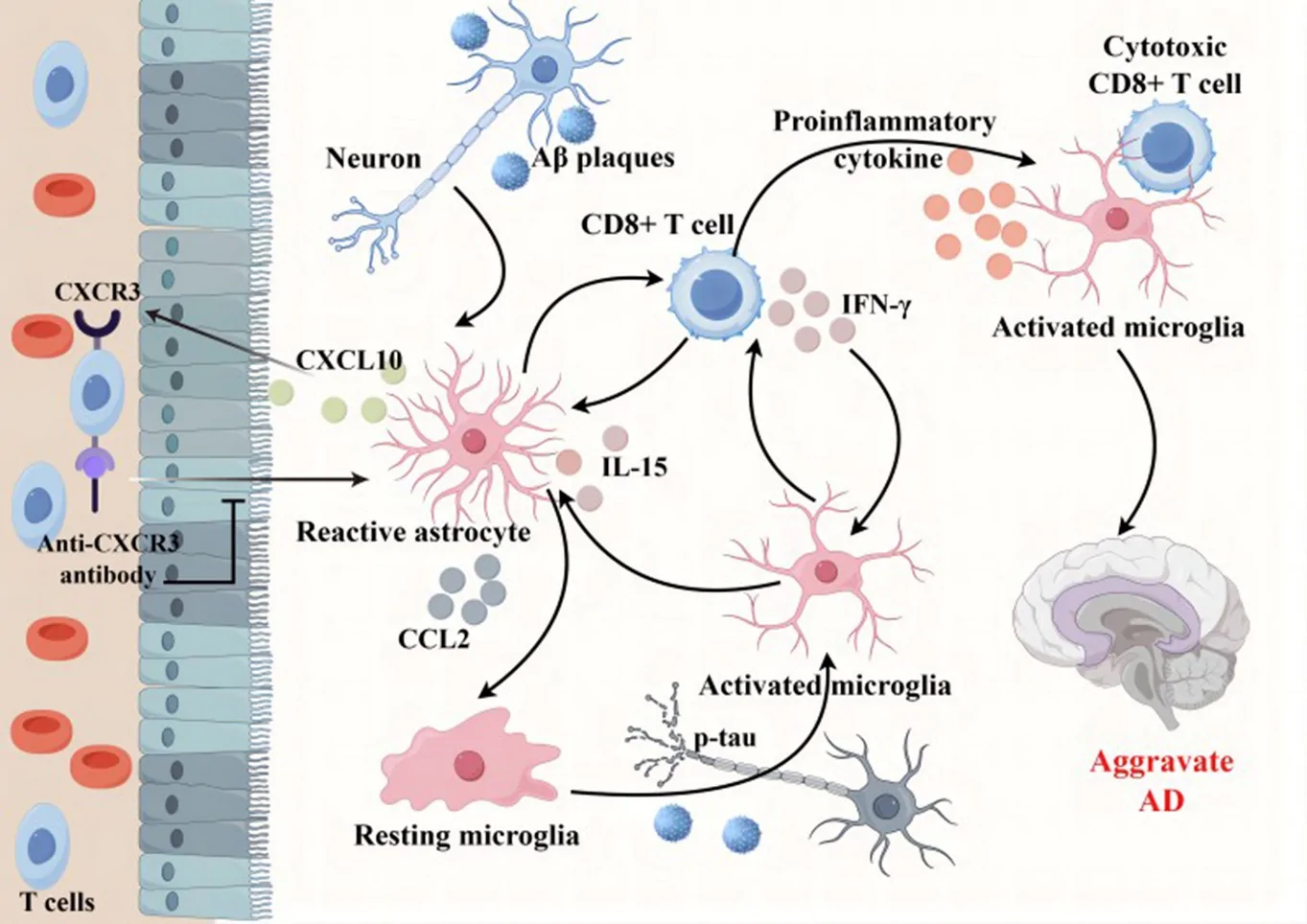
**The potential mechanism of action of CD8 cells in AD**. The pathology of AD results in elevated levels of proinflammatory chemokines. When CXCL10 binds to the T-cell receptor CXCR3, T cells specifically migrate into the diseased environment of AD. Additionally, CCL2 interacts with CCR2 to stimulate the recruitment of microglia. The infiltrating CD8^+^ T cells then activate microglia, further worsening cell damage and neuroinflammation in AD.

Another recent study provided further evidence supporting the notion that the frequency of CD8^+^ T_EMRA_ cells, a marker of T-cell aging, is elevated in both the peripheral blood and CSF of AD patients. This increase is inversely correlated with cognitive function [63, 201]. Senescent T cells exhibit impaired proliferation in response to TCR stimulation and secrete various inflammatory factors as part of the SASP (senescence-associated secretory phenotype). These cells are characterized by a lack of costimulatory receptors CD27 and CD28, along with an upregulation of NK cells related markers [202]. A key characteristic of aging in the nervous system is a reduction in both the volume and function of white matter; however, the specific mechanisms that lead to white matter pathology remain unclear. Using single-cell RNA sequencing, Mikeal et al. [203] identified a type of oligodendrocyte that responds to IFN and found that these cells are located in close proximity to CD8^+^ T cells within aging white matter [203]. Ultimately, their findings suggested that CD8^+^ T-cell-induced IFN responsive oligodendrocytes (ODCs) and microglia play a significant role in modifying the aging process of white matter [203]. CD8^+^ T cells can directly target neurons and their neurites or indirectly cause 'collateral' neuronal damage after destroying the myelin sheath and ODCs in both the gray and white matter of the CNS [203]. Heinz et al. [204] utilized time-lapse video microscopy and two-photon imaging alongside whole-cell patch-clamp recordings to show two distinct functional outcomes when antigen-presenting neurons directly interacted with antigen-specific CD8^+^ T cells. This study emphasized that electrical silencing is an immediate result of MHC I-restricted interactions between CD8^+^ T cells and neurons [205-208]. The potential mechanism of action of CD8^+^ T cells in AD is elaborated on in Figure 3.

## 5. Discussion

A growing body of evidence now suggests that systemic inflammation can impact the development and progression of AD [209]. Recently, there has been a focus on adaptive immunity as a potential new target for AD treatment. Clinical trials testing AD vaccines are underway, highlighting the significant role of peripheral immune activity in AD [210]. The intricate characteristics of the CNS require unique immunological adjustments to identify and react to shifts in the environment. In stable conditions, microglia along with various innate immune cells inhabit and monitor the brain's parenchyma, assisting in the clearance of cellular debris and preserving the local microenvironment. This process is probably essential for the proper functioning of synapses and overall connectivity. The intact BBB, meningeal layers, CSF and interstitial fluid (ISF) flow, along with the dural lymphatic system, collaboratively function to protect the brain as a relatively immune-privileged organ. This system also acts as a reservoir for immune cells, facilitating communication between the peripheral immune system and brain parenchyma. Dysfunction of these protective structures may result in the ectopic infiltration of peripheral immune cells, particularly those of the adaptive immune system, into the brain parenchyma, thereby creating a novel immune environment in AD. In a healthy brain, the entry of peripheral immune cells is carefully regulated to maintain balance. However, during neuroinflammatory conditions, immune responses within and outside the CNS lead to the infiltration of CNS antigen-specific T cells from peripheral lymph nodes into the brain [211]. Interestingly, Abeta antigens in the brain can travel to peripheral lymph nodes and be presented to T cells by APCs in the periphery [212]. These events may trigger peripheral adaptive immune activation, particularly in the early stages of AD [132, 211, 213]. As the disease progresses, dysfunction in meningeal lymphatic function can block Abeta drainage and lead to Abeta accumulation in the meninges [214]. Functional lymphatic vessels are present within the dura, capable of transporting fluid and cytokines from the CSF that exits the brain parenchyma, as well as local immune cells, such as T cells, to the dCLN. This transport facilitates interactions between brain-derived antigens and peripheral T cells [215-217]. The discovery of the dural lymphatic system has provided new insights into the communication mechanisms between the CNS, its border zones, and the periphery, particularly concerning neuroimmune interactions. These findings may illuminate the roles of neuroimmune factors in neurodegenerative diseases. Meningeal lymphatic dysfunction was observed in 13- to 14-month-old 5×FAD mice, which exhibit significant Abeta deposition throughout the meninges. The ablation of meningeal lymphatics in these 5×FAD mice exacerbated Abeta deposition in both the brain and meninges, leading to increased microgliosis and neurovascular dysfunction, and adversely affecting the outcomes of anti-Abeta immunotherapy. Notably, enhancing meningeal lymphatic function in conjunction with Abeta antibody immunotherapy resulted in improved Abeta clearance from the brain [214, 218]. Future research should focus on the critical question of how meningeal lymphatic endothelial cells, along with other cellular components of the meningeal lymphatic vessels in the human CNS, are simultaneously influenced by AD-related pathologies. Additionally, it is important to explore whether targeting the brain lymphatic system can effectively modulate neuroimmune interactions to impact tauopathy and tau-mediated neurodegeneration.

Accumulating genetic and functional evidence strongly suggests that adaptive immunity plays both beneficial and detrimental roles in the pathogenesis and progression of AD. However, it remains unclear whether these effects are direct or indirect. The genetic removal of peripheral immune cell types in Rag^-/-^-5×FAD mice, who were deficient in T cells, B cells, NK cells, and NKT cells, markedly accelerated Abeta pathology, intensified neuroinflammation, raised the number of microglia, reduced the branching of microglial processes, and diminished microglial phagocytic activity [219]. Conversely, administering IgG or performing bone marrow transplantation reversed these changes and lowered Abeta pathology, indicating that peripheral adaptive immunity plays a role in the development of AD [219]. In another study, fibrillar Abeta deposits and total Abeta levels were significantly reduced in Rag2^-/-^-APPswe/PSEN1dE9 mice. Furthermore, microgliosis and Abeta phagocytosis were enhanced following the reconstitution of APPswe/PSEN1dE9 mice with Rag2^-/-^ bone marrow. These findings suggest that both lifelong and acquired absence of T and B cells is associated with decreased Abeta pathology [220]. Nonetheless, neuronal loss was also found in the hippocampus of individuals with non-AD, while T cells did not show a consistent correlation with plaques or NFTs [62]. In a separate investigation, researchers noted the presence of CD8^+^ T cells in the brains of AD patients, particularly within the hippocampus, where tau pathology was present but Abeta pathology was absent [88]. These observations suggest that T cells may play a significant role in the progression of AD; however, the presence of T cells within the brain's parenchyma necessitates a complex interplay of disease and immune factors.

Despite significant advancements in our understanding of AD pathophysiology and the development of potential therapeutic interventions, particularly those targeting Abeta, the fundamental mechanisms underlying the clinically symptomatic phase of AD remain elusive. A more comprehensive understanding of the specific roles of both the innate and adaptive immune systems throughout the progression of AD pathology, especially during the tau phase, is likely to facilitate the development of novel therapeutic strategies for both the pre-clinical and clinically symptomatic phases of AD. In addition, there is a bidirectional communication pathway between the peripheral immune system and the brain in AD. This pathway may include various interactions, such as vagal sensory afferents, brain-derived substances entering the bloodstream and affecting peripheral immune cells, communication between peripheral immune cells or active factors and microglia in the brain, and direct infiltration of peripheral immune cells into the brain tissue. The deposition of Abeta and tau aggregates in AD leads to activation of the brain's immune system, damage to the BBB, and infiltration of peripheral immune cells into brain tissue, altering the innate and adaptive immune environment. Thus, mapping the interconnections between innate and adaptive immunity in a disease-state-specific context, particularly focusing on microglia and T cells, is crucial for understanding their communication mechanisms, antigen presentation, and pathophysiological responses. This knowledge will serve as a key nexus for developing unique therapeutic interventions aimed at preventing or reversing neurodegeneration in AD. While the exact mechanism of AD onset remains unclear, and current treatments are not entirely effective. A better understanding of the roles of adaptive immunity within the context of the CNS immune milieu will enhance our comprehension of how adaptive immune effectors, along with the interactions between innate and adaptive immune cells, contribute to the development and progression of AD and innovative therapeutic strategies for clinical management.
